# Evidence Use and Identifier-Conditioned Prior Knowledge in Large Language Model Classification of Oncology Trials Assessed Through Progressive Content Removal and Counterfactual Testing: Comparative Analysis

**DOI:** 10.2196/95565

**Published:** 2026-07-29

**Authors:** Paul Windisch, Carole Koechli, Fabio Dennstädt, Daniel M Aebersold, Daniel R Zwahlen, Robert Förster, Christina Schröder

**Affiliations:** 1 Department of Radiation Oncology, Kantonsspital Winterthur, Brauerstrasse 15, Winterthur, Zurich, 8401, Switzerland, 41 052 266 26 53; 2 Department of Radiation Oncology, Inselspital, Bern University Hospital, University of Bern, Bern, Switzerland

**Keywords:** natural language processing, large language models, memory, reasoning models, context grounding, parametric knowledge

## Abstract

**Background:**

Large language models (LLMs) can accurately classify biomedical documents, but strong benchmark performance does not establish that predictions are grounded in the supplied text. In biomedical literature tasks, titles, abstracts, digital object identifiers (DOIs), journal metadata, and trial identifiers may have been seen during pretraining and can trigger parametric knowledge or learned associations.

**Objective:**

This study aimed to test whether oncology randomized trial success classification is driven by abstract evidence or by identifier-conditioned prior knowledge, and assess whether models follow counterfactual outcome evidence when it conflicts with original trial identifiers.

**Methods:**

We evaluated 250 two-arm oncology randomized controlled trials from 7 major journals published between 2005 and 2023, each with a single primary endpoint and previously adjudicated positive or negative ground-truth label. The corpus included 58.4% (146/250) positive and 41.6% (104/250) negative trials. GPT-5.2, Gemini 3 Flash, and Claude Opus 4.5 were queried via vendor APIs under default settings using a single-token output instruction. For each trial, we created 5 deterministic input conditions: title+abstract, title only, DOI only, counterfactual title+abstract in which the primary endpoint outcome statement was minimally flipped, and the same counterfactual input paired with the original DOI to create an identifier-text conflict. Performance was assessed using valid format rate, accuracy, sensitivity, specificity, and *F*_1_-score.

**Results:**

The models showed high format adherence, with valid prediction rates of 97.2% to 100%. In the title+abstract condition, all models achieved high and balanced performance (accuracy and *F*_1_-score=0.96-0.97; sensitivity=0.96-0.97; specificity=0.96-0.98). Removing evidence reduced performance stepwise: title-only accuracy and *F*_1_-score fell to 0.79 to 0.88, and DOI-only performance fell to 0.63-0.67, exceeding the 58.4% majority class baseline but indicating limited identifier-driven signal. Counterfactual edits were concentrated in outcome-bearing text, with the Results and Conclusions sections modified for all trials, whereas the titles and Methods sections required edits in only 5.2% (13/250) and 1.6% (4/250) of trials. Against inverted labels, models followed counterfactual evidence with near-ceiling performance (accuracy and *F*_1_-score=0.96-0.99). Reintroducing the original DOI caused little change for GPT-5.2 (accuracy and *F*_1_-score=0.99) but modestly reduced *F*_1_-scores for Gemini (0.97) and Claude (0.95), mainly through lower sensitivity.

**Conclusions:**

The evaluated LLMs robustly followed explicit end point statements in abstracts, including when those statements contradicted original trial outcomes. However, above-chance title-only and DOI-only performance, together with small decrements under counterfactual DOI conflicts, showed that identifiers can carry predictive signal and occasionally compete with textual evidence. Progressive content removal combined with counterfactual identifier-text conflicts offers a practical, reproducible audit for grounding in biomedical LLM evaluations.

## Introduction

Large language models (LLMs) are increasingly used for biomedical text processing, including automated structuring of electronic health records, clinical trial matching, and literature screening in evidence synthesis workflows [1-3].

However, LLM success on biomedical text tasks is not by itself evidence that the output is grounded in the provided input. A defining feature of modern LLMs is that they store substantial “parametric knowledge” acquired during pretraining, and in some settings, they can behave like implicit knowledge bases that retrieve facts from model weights rather than from the prompt context [4]. In medicine specifically, LLMs have been shown to encode clinically relevant knowledge and perform strongly on medical question answering benchmarks, reinforcing that model parameters can serve as a powerful source of prior information independent of any supplied document [5]. For biomedical publishing and evidence synthesis, this distinction is consequential: users may expect the system to follow the text in front of it, whereas the model may instead draw on associations encoded during training with the underlying paper, trial name, venue, or related metadata.

Concerns about noncontextual sources of task performance are amplified by 2 related phenomena: memorization and data contamination. Previous work has demonstrated that LLMs can leak or reproduce training data under targeted querying, indicating that verbatim or near-verbatim sequences can be memorized and later elicited [6]. More recent work has developed formal measures for quantifying extractability and memorization risk, highlighting that the phenomenon can be measured and varies with model and inference choices [7]. Separately, systematic analyses have documented that benchmark contamination is widespread in the LLM era and can occur at nontrivial rates, creating the possibility that models appear to “solve” tasks by recognizing previously seen evaluation items rather than generalizing [8]. In biomedical domains, where many inputs (titles, abstracts, and digital object identifiers [DOIs]) are publicly available and plausibly included in pretraining corpora, a model might correctly predict a randomized controlled trial’s (RCT) outcome without relying primarily on the outcome evidence contained in the abstract.

A growing methodological literature has therefore begun to explicitly separate contextual evidence use from prior (parametric) knowledge. Work on knowledge conflicts shows that language models do not integrate context and prior knowledge uniformly and may privilege prior knowledge when the model is “familiar” with the entity or topic referenced [9]. Methods such as contrastive decoding have been proposed to reduce overreliance on encoded priors and improve the use of contextual information when the prompt provides relevant evidence [10]. Relatedly, counterfactual paradigms have been used to disentangle parametric from contextual knowledge by constructing prompts in which the provided context is intentionally altered, enabling direct tests of whether a model follows the prompt or defaults to internal memory [11]. In the biomedical setting, dedicated benchmarks have further emphasized that knowledge conflicts and unfaithful grounding are practical risks when users provide incomplete, contradictory, or incorrect context [12]. Despite these advances, biomedical evaluations that use real documents rarely operationalize a direct, auditably reproducible test of context-grounded evidence use vs identifier-conditioned signal that isolates the informational contribution of the input text from that of identifiers.

Recent clinical LLM studies similarly caution that apparent medical performance may reflect brittle pattern use or unfaithful grounding rather than reliable use of case-specific evidence. Bedi et al [13] showed that medical reasoning benchmark performance can change when standard answer structures are perturbed, supporting the need to distinguish reasoning from recognition. Omar et al [14] demonstrated that clinical decision support outputs are vulnerable to adversarial hallucination attacks, with models often elaborating fabricated prompt details despite mitigation attempts. Together with broader calls for task-specific validation before clinical adoption [15], these findings motivate evaluations that explicitly test whether models follow supplied biomedical evidence when it conflicts with prior associations.

This study focused on that methodological gap using the concrete task of classifying oncology RCTs as positive vs negative with respect to primary end point attainment.

To distinguish context-grounded task performance from identifier-driven prior signal, we leveraged an evaluation framework based on progressive content removal and counterfactual results. The key intuition was diagnostic: if performance remained meaningfully above chance when only identifiers were provided, this would indicate that identifiers carry a predictive signal for the model, potentially through memorized associations, learned metadata correlations, or recognition of trial-specific cues. If predictions then failed to flip under counterfactual results text, then the model’s prediction pattern would be more consistent with identifier-conditioned prior signal than with reliance on the supplied outcome evidence.

We evaluated 3 widely used commercial models under default settings to reflect typical end user deployment conditions. By combining progressive ablations with counterfactual conflict tests, this study aimed to provide an empirically grounded answer to a practical question that is often implicit in biomedical LLM benchmarking: when an LLM classifies a clinical trial abstract correctly, how much of that performance is attributable to the supplied text vs identifier-conditioned prior information?

## Methods

This study evaluated whether LLM performance on biomedical trial success classification is primarily driven by information contained in the provided input text or by recognition, parametric knowledge, and learned associations with identifiers.

### Data and Annotation

We used an existing dataset that was used in a previous paper from our group consisting of 250 RCTs drawn from 7 major medical journals (*British Medical Journal*, *JAMA*, *JAMA Oncology*, *Journal of Clinical Oncology*, *The Lancet*, *The Lancet Oncology*, and *New England Journal of Medicine*) published between 2005 and 2023 [16]. In the aforementioned publications, eligible trials were restricted to designs with exactly 2 arms and a single primary end point, and abstracts were retrieved via PubMed and parsed from text to create the study corpus. For the present analysis, we reused the released dataset and corresponding ground-truth labels.

Ground-truth labels were adopted from the original dual-annotation procedure performed by 2 authors (PW and CK), in which trials were classified as positive if the primary end point was met and as negative otherwise. The annotation workflow included an initial calibration phase followed by independent labeling and consensus resolution of discrepancies. Full texts or protocols were consulted only when the abstract did not clearly report the primary end point and its results. We did not modify labels or repeat manual annotation for the present analysis.

### Experimental Conditions

To distinguish evidence use from identifier-driven recognition, we generated 5 input variants for each RCT. The baseline condition contained the trial title and abstract (title+abstract). A title-only condition contained only the trial title. A DOI-only condition contained only the DOI string. A counterfactual “fake results” condition contained the potentially edited title and an edited abstract in which the reported primary end point outcome was flipped (positive to negative or negative to positive) while keeping all other content maximally unchanged. Counterfactual abstracts were created by identifying the sentences reporting primary end point attainment (typically in the Results and/or Conclusions sections) and minimally editing the outcome language (eg, reversing whether the primary end point was met or reversing the direction of statistical significance statements tied to the primary end point). Background, design, population, interventions, and secondary outcome text were left unchanged unless modification was required to maintain internal coherence. The original and counterfactual texts are provided in the GitHub repository that is referenced in the Data Availability section. Finally, we input the counterfactual title and abstract together with the original DOI to create a direct conflict between the supplied counterfactual evidence and identifier-conditioned prior information.

The task for the LLMs was to determine, from the provided information, whether the trial met its primary end point. While this is essentially impossible from a DOI with the exception of a slight bias of high-impact journals to publish positive trials, titles sometimes report results and usually those related to the primary end point. Abstracts usually but not always state which end point was the primary one and explicitly provide the results per end point. To align with the previously published workflow and minimize ambiguity, we used an explicit instruction format requiring the model to output exactly 1 token-level label: “POSITIVE” or “NEGATIVE,” all capitalized.

### Models and Prompts

The following system prompt was used: “You will be provided with information about a randomized controlled oncology trial. Your task will be to classify if the trial was positive, i.e. if it met its primary endpoint, or negative, i.e. if it did not meet its primary endpoint. Your response should be either the word POSITIVE (in all caps) or NEGATIVE (in all caps). Do not output anything else.” The user prompt consisted of the respective input variant (title+abstract, title only, DOI only, counterfactual title+abstract, and DOI and counterfactual title+abstract).

We did not use few-shot examples, retrieval augmentation, or chain-of-thought prompting as the aim was to evaluate default single-label behavior rather than prompt-engineered mitigation strategies.

Three commercial LLMs were evaluated via their vendor APIs in a local pipeline (Claude [Anthropic], Google Gemini, and GPT-5.2 [OpenAI]). The evaluated model IDs were gpt-5.2-2025-12-11, gemini-3-flash-preview, and claude-opus-4-5-20251101. Models were queried under default vendor settings to reflect typical end user deployment conditions. No additional decoding parameters were set, and no seed was specified. No vendor-specific reasoning control was requested.

Responses were considered valid only if the returned text matched exactly the “POSITIVE” or “NEGATIVE” label after trimming white space. Any other output was recorded as invalid for downstream summaries.

### Statistical Analysis

The primary objective was to quantify how model behavior changed as informational content was removed and when counterfactual results created a direct conflict between the provided abstract and the original trial outcome potentially associated with the identifier. Performance for the baseline, title-only, and DOI-only conditions was summarized using *F*_1_-score (and accuracy) against the original ground-truth label. For the counterfactual condition, the expected label was defined as the inverse of the original ground truth, and performance was evaluated against this counterfactual target. In addition to per-condition performance, we quantified counterfactual sensitivity by calculating the proportion of trials for which the predicted label differed between the baseline and counterfactual inputs (flip rate), and we summarized invalid-format outputs per model and condition. For the calculation of the *F*_1_-score, only valid (ie, correctly formatted predictions) were considered. The 95% CIs were estimated using normal approximation intervals. We did not analyze token-level probabilities or calibration because comparable logits were not consistently available across the evaluated vendor APIs.

### Ethical Considerations

This study used publicly available abstracts from published clinical trials. It did not involve human beings, material of human origin, health-related personal data, deceased persons, or human embryos. Therefore, per the applicable regulation (ie, the Swiss Human Research Act and its ordinances), ethics approval was not required [17].

## Results

**Figure 1.**
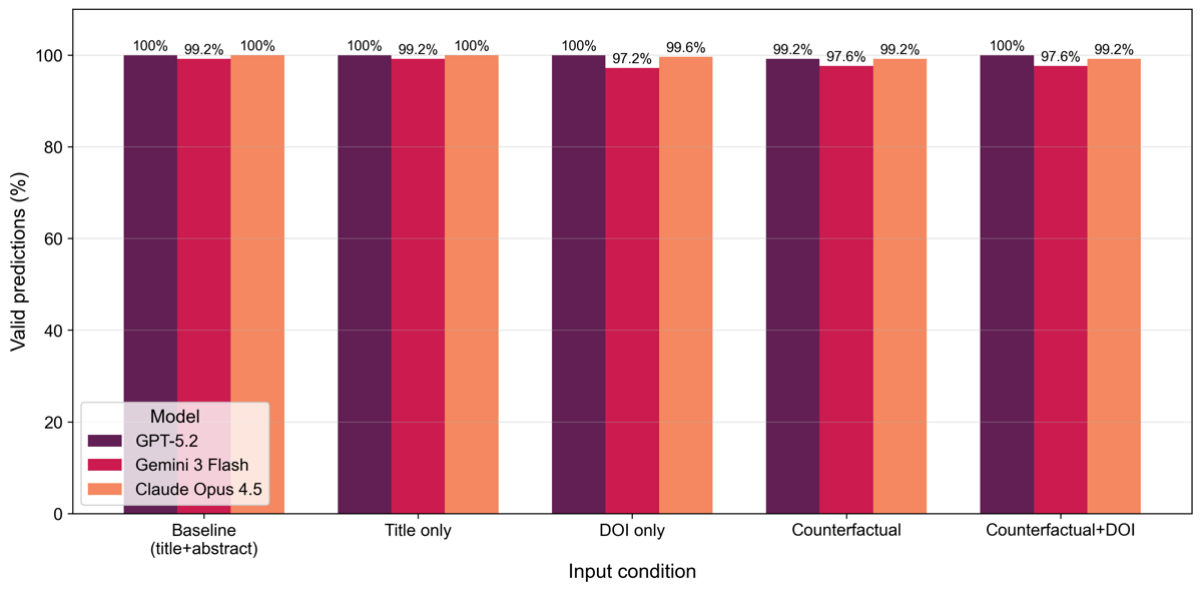
Valid prediction rates of GPT-5.2, Gemini 3 Flash, and Claude Opus 4.5 under different conditions. DOI: digital object identifier.

Across conditions, the models adhered closely to the required single-token response format (Table 1). Valid prediction rates ranged from 97.2% to 100% depending on model and condition (Figure 1). GPT-5.2 returned 100% valid predictions across all but one condition. Minor format deviations were observed mainly for Gemini (eg, 97.2% valid in the DOI-only condition and 97.6% valid in the counterfactual setting), whereas Claude remained near ceiling (99.2%-100%) across all conditions.

**Table 1.** Performance of GPT-5.2, Gemini 3 Flash, and Claude Opus 4.5 under different conditions.

Condition and model	Valid predictions (%)	Accuracy (95% CI)	Sensitivity (95% CI)	Specificity (95% CI)	*F*_1_-score (95% CI)
Baseline					
GPT-5.2	100	0.96 (0.94-0.99)	0.97 (0.94-1.00)	0.96 (0.92-1.00)	0.96 (0.94-0.99)
Gemini 3 Flash	99.2	0.96 (0.94-0.99)	0.96 (0.93-0.99)	0.97 (0.94-1.00)	0.96 (0.94-0.99)
Claude Opus 4.5	100	0.97 (0.95-0.99)	0.97 (0.94-1.00)	0.98 (0.95-1.00)	0.97 (0.95-0.99)
Title only					
GPT-5.2	100	0.82 (0.78-0.87)	0.82 (0.75-0.88)	0.84 (0.77-0.91)	0.82 (0.77-0.87)
Gemini 3 Flash	99.2	0.88 (0.84-0.92)	0.88 (0.83-0.93)	0.88 (0.81-0.94)	0.88 (0.84-0.92)
Claude Opus 4.5	100	0.79 (0.74-0.84)	0.79 (0.73-0.86)	0.78 (0.70-0.86)	0.78 (0.73-0.83)
DOI^a^ only					
GPT-5.2	100	0.67 (0.61-0.73)	0.76 (0.69-0.83)	0.55 (0.45-0.64)	0.66 (0.60-0.71)
Gemini 3 Flash	97.2	0.63 (0.57-0.69)	0.55 (0.47-0.63)	0.75 (0.67-0.84)	0.63 (0.57-0.69)
Claude Opus 4.5	99.6	0.63 (0.57-0.69)	0.63 (0.55-0.71)	0.63 (0.54-0.73)	0.63 (0.57-0.69)
Counterfactual					
GPT-5.2	99.2	0.99 (0.98-1.00)	0.99 (0.97-1.00)	0.99 (0.98-1.00)	0.99 (0.98-1.00)
Gemini 3 Flash	97.6	0.98 (0.96-1.00)	0.96 (0.92-1.00)	0.99 (0.98-1.00)	0.98 (0.96-1.00)
Claude Opus 4.5	99.2	0.96 (0.94-0.99)	0.92 (0.87-0.97)	0.99 (0.98-1.00)	0.96 (0.94-0.99)
Counterfactual+DOI					
GPT-5.2	100	0.99 (0.97-1.00)	0.99 (0.97-1.00)	0.99 (0.97-1.00)	0.99 (0.97-1.00)
Gemini 3 Flash	97.6	0.97 (0.94-0.99)	0.92 (0.87-0.97)	1.00 (1.00-1.00)	0.97 (0.94-0.99)
Claude Opus 4.5	99.2	0.95 (0.92-0.98)	0.90 (0.84-0.96)	0.98 (0.96-1.00)	0.95 (0.92-0.97)

a DOI: digital object identifier.

**Figure 2.**
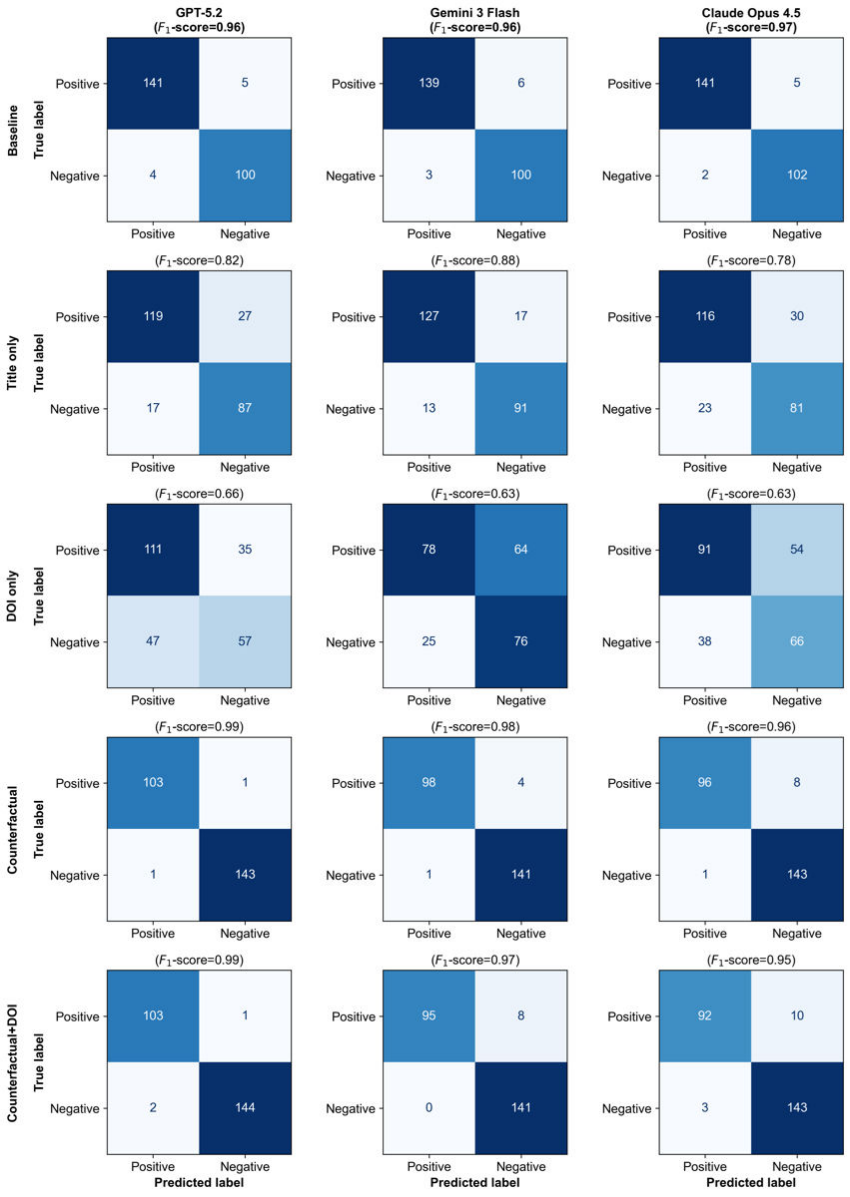
Confusion matrices of GPT-5.2, Gemini 3 Flash, and Claude Opus 4.5 under different conditions. DOI: digital object identifier.

The confusion matrices for all models and conditions are provided in Figure 2. The corpus of 250 trials contained 146 (58.4%) positive trials and 104 (41.6%) negative trials; always predicting the majority class would yield a 58.4% accuracy and a macro–*F*_1_-score of 0.37. When provided with the full title and abstract, all 3 models achieved high and tightly clustered classification performance (accuracy=0.96-0.97; *F*_1_-score=0.96-0.97; Table 1). Sensitivity and specificity were similarly high (sensitivity=0.96-0.97; specificity=0.96-0.98), indicating balanced performance for positive and negative trials when the complete abstract text was available.

**Figure 3.**
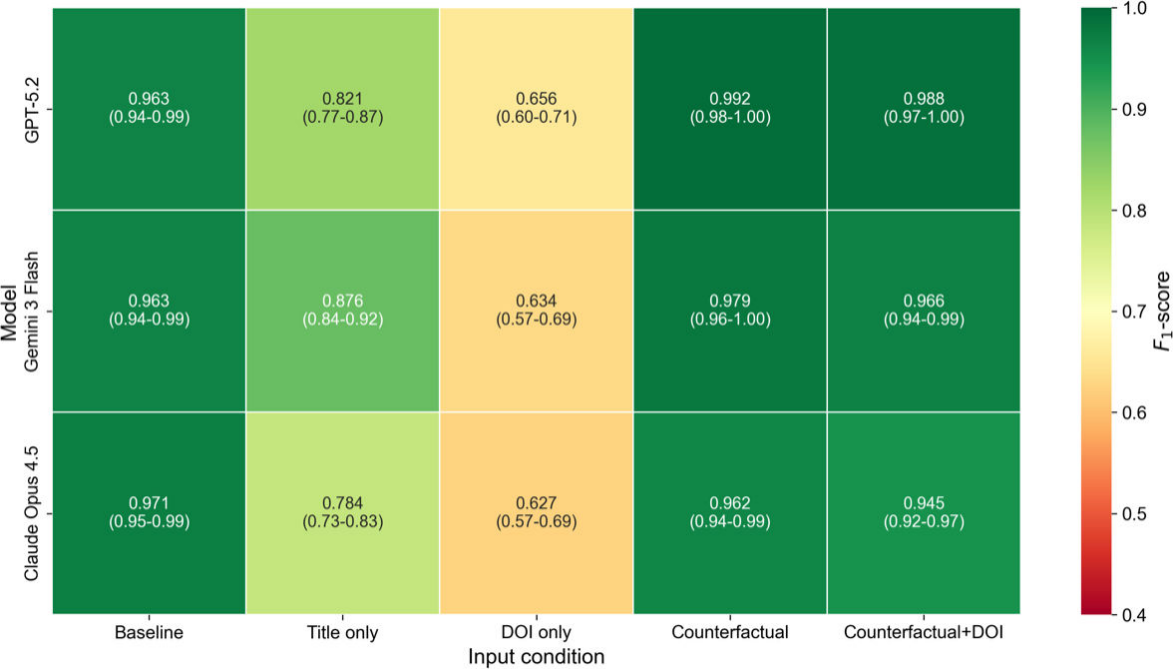
Heat map of the *F*_1_-scores of GPT-5.2, Gemini 3 Flash, and Claude Opus 4.5 under different conditions. The numbers in parentheses indicate the 95% CIs. DOI: digital object identifier.

Removing abstract content reduced performance in a stepwise manner. Under the title-only condition, accuracy fell to 0.79 to 0.88, and the *F*_1_-score fell to 0.78 to 0.88, with Gemini 3 Flash performing best (accuracy and *F*_1_-score=0.88) and Claude Opus 4.5 performing worst (accuracy=0.79; *F*_1_-score=0.78). Sensitivity and specificity remained broadly comparable within each model (GPT-5.2=0.82 vs 0.84; Gemini=0.88 vs 0.88; Claude=0.79 vs 0.78, respectively), suggesting that performance degradation with titles alone was not driven solely by one-sided misclassification. With DOI-only inputs, performance decreased further for all models (accuracy=0.63-0.67; *F*_1_-score=0.63-0.66). Error profiles diverged by model: GPT-5.2 showed higher sensitivity than specificity (0.76 vs 0.55), whereas Gemini showed the reverse pattern (0.55 vs 0.75), and Claude remained relatively symmetric (0.63 vs 0.63). The *F*_1_-scores for all models and conditions are visualized in Figure 3.

To generate counterfactual abstracts, edits were concentrated in outcome-bearing portions of the text. Across 100% (250/250) of the trials, both the Results and Conclusions sections required modification. In contrast, edits were rarely required in the title (13/250, 5.2%) or Methods section (4/250, 1.6%), and the Introduction section did not require modification (0%).

Against the inverted ground-truth labels, the models followed the counterfactual evidence with near-ceiling performance. In the counterfactual condition, accuracy and *F*_1_-score ranged from 0.96 to 0.99 (GPT-5.2=0.99; Gemini=0.98; Claude=0.96). Specificity remained high (0.99 for all 3 models), whereas Claude’s decrease relative to GPT-5.2 and Gemini was driven mainly by lower sensitivity (0.92, 95% CI 0.87-0.97).

When the original identifier was reintroduced (counterfactual+DOI), performance changed minimally for GPT-5.2 (accuracy and *F*_1_-score=0.99) but decreased modestly for Gemini (accuracy and *F*_1_-score=0.97) and Claude (accuracy and *F*_1_-score=0.95). Notably, Gemini maintained perfect specificity (1.00) in this condition (95% CI 1.00-1.00) alongside reduced sensitivity (0.92), whereas Claude showed a larger sensitivity drop (0.90) with specificity still high (0.98).

Information on additional input conditions (abstract only, title+DOI, and counterfactual title only), as well as examples of counterfactual titles and abstracts, can be found in Multimedia Appendix 1.

## Discussion

### Principal Findings

All 3 commercial models achieved high performance when given full title and abstract information (accuracy and *F*_1_-score clustered around 0.96-0.97), with only minor rates of invalid-format outputs. Performance decreased stepwise as informational content was removed: with title-only inputs, accuracy dropped to a range of 0.79 to 0.88, and with DOI-only inputs, it fell further to a range of 0.63 to 0.67. In contrast, when the abstract’s primary outcome statement was counterfactually flipped, the models followed the altered evidence and achieved near-ceiling performance against the inverted labels (accuracy and *F*_1_-score=0.96-0.99). Reintroducing the real DOI alongside the counterfactual abstract minimally affected GPT-5.2 but led to modest performance reductions for Gemini and Claude, primarily via reduced sensitivity, suggesting occasional identifier-driven interference when DOI and results text conflicted.

### Interpretation and Comparison to Prior Work

Interpreting these results in the context of prior work, the strong baseline performance is consistent with broader evidence that LLMs can perform competitively on biomedical language tasks [1,3]. However, high task performance alone does not establish that the prediction is grounded in the provided document rather than influenced by parametric knowledge or learned associations, an issue long discussed in the framing of language models as implicit knowledge bases [4]. The stepwise ablation results speak directly to that distinction. Title-only performance well above chance can plausibly arise from a mixture of (1) genuine, weakly informative cues in titles (eg, disease setting, intervention class, and end point hints); and (2) recognition of well-known trials from pretraining exposure. DOI-only inputs are informative because the DOI string contains minimal semantic content for end point attainment. Above-chance performance under DOI-only conditions is therefore compatible with identifier-triggered recall, learned metadata correlations, or other DOI-associated regularities.

However, the counterfactual results indicate that the models’ predictions were usually dominated by the supplied counterfactual outcome evidence rather than by any identifier-associated prior signal. When the outcome-bearing sentences in the abstract were minimally edited to flip the primary end point conclusion, the models overwhelmingly produced the inverted label, indicating sensitivity to the supplied text even when that text contradicted the original outcome potentially associated with the trial identifier. At the same time, the small but consistent decrement for some models when the real DOI was appended to the counterfactual abstract suggests that identifiers can still exert a measurable pull on the model’s decision, echoing findings that models do not integrate context and prior knowledge uniformly and can privilege priors when they are “familiar” with an entity [9]. It is important to emphasize that our counterfactual flips created direct, high-salience evidence conflicts. In more realistic settings, users may supply incomplete or subtly contradictory evidence, where LLMs can struggle with conflict resolution in biomedical contexts [12]. Taken together, the findings support a nuanced conclusion: the models can robustly follow explicit outcome statements in abstracts (strong context-grounded behavior under explicit outcome evidence) yet still display some degree of susceptibility to identifier-driven priors when content is sparse or when identifiers are introduced.

### Strengths and Limitations

Several strengths support the interpretability and practical relevance of these findings. First, the design operationalizes the contrast between context-grounded evidence use and identifier-conditioned prior signal using deterministic, logged perturbations (progressive content removal plus counterfactual outcome edits), which makes the diagnostic logic transparent and reproducible. Second, the evaluation used a corpus of real oncology RCT abstracts spanning multiple high-impact journals and many publication years, aligning the test distribution with how LLMs are used in evidence synthesis and clinical research workflows rather than relying solely on synthetic benchmarks. Third, the single-token output constraint (“POSITIVE” and “NEGATIVE”) reduced ambiguity in scoring and minimized the confounding role of verbose explanations, whereas the high valid output rates indicate that the API-based setup was stable across conditions. Fourth, comparing multiple commercial models under default settings improved external validity for typical end user deployment, where practitioners rarely tune decoding parameters or implement specialized grounding interventions.

Several limitations should temper overgeneralization. First, this study focused on a single binary task (primary end point met vs not met) within oncology RCTs. The balance between context-grounded evidence use and identifier-conditioned prior signal may differ for tasks requiring finer-grained extraction, multi-label judgments, or synthesis across multiple documents. Second, DOI-only above-chance performance could partly reflect indirect correlations encoded in DOI structure (publisher prefix, journal family, and year) rather than memorized trial-specific outcome associations. Additional controls (eg, DOI shuffling across papers and synthetic DOIs matched on prefix and year) would help isolate the mechanism. Third, counterfactual editing was designed to be minimal, but any manual or rule-based editing procedure risks introducing artifacts (lexical cues, unnatural phrasing, or consistency breaks) that models could exploit. Even though edits were concentrated where outcomes were stated, future work could quantify the detectability of edits or use blinded human review to ensure counterfactual naturalness. Fourth, the models were queried via vendor APIs with default settings and without a fixed seed. While this reflects realistic use, it limits ANOVA due to decoding stochasticity and complicates strict reproducibility across future model snapshots. Finally, we did not evaluate model calibration, abstention behavior, or uncertainty reporting—properties that may be crucial when deploying such systems in safety-critical evidence workflows.

### Outlook

Future research could try to strengthen causal attribution of identifier effects by adding negative controls: swapping DOIs between trials, adding semantically irrelevant identifiers, or using “matched” synthetic DOIs, which would help separate true memorized mapping from metadata correlations. A second direction is to broaden task coverage: applying the same progressive ablation and counterfactual conflict framework to other biomedical natural language processing tasks (population, intervention, comparator, and outcome extraction; effect direction and magnitude; toxicity end points; and comparative effectiveness statements) would test whether strong counterfactual sensitivity generalizes beyond this relatively explicit classification problem. A third direction is temporal and contamination-robust evaluation: constructing time-split test sets consisting of trials published after known model training cutoffs (or using newly published or embargoed material where feasible) would reduce the plausibility of trial-specific parametric associations and better isolate context-grounded task performance.

### Conclusions

In conclusion, this study suggests that the evaluated models were highly sensitive to explicit outcome statements in oncology RCT abstracts, as shown by near-ceiling performance on counterfactual abstracts that directly contradicted the original results. At the same time, above-chance performance with titles—and especially with DOI-only inputs—indicates that identifiers can carry predictive signal for these models, consistent with identifier-associated predictive signal that is at least partly independent of the provided abstract text. The modest degradation in performance observed when reintroducing real DOIs into counterfactual abstracts further implies that identifier-triggered priors can occasionally compete with textual evidence.

## Supplementary material

10.2196/95565 Multimedia Appendix 1 Supplementary material containing representative counterfactual trial examples and additional input conditions.

## Abbreviations

DOI: digital object identifier
LLM: large language model
RCT: randomized controlled trial
