# Supplementary material for: Evidence Use and Identifier-Conditioned Prior Knowledge in Large Language Model Classification of Oncology Trials Assessed Through Progressive Content Removal and Counterfactual Testing: Comparative Analysis
Source: JMIR AI. 2026 Jul 29;5:e95565. doi: 10.2196/95565 (PMC13419288; doi:10.2196/95565)
Supplement: Multimedia Appendix 1 [file ai-v5-e95565-s001.docx]

**Supplement part 1: Representative Counterfactual Trial Examples**

**Label definition.** The original gold label indicates whether the primary endpoint was met in the source trial. The counterfactual gold label is the inverted target label after outcome-bearing text was minimally edited while preserving the trial design, population, interventions, and other non-outcome context where possible.

| **Example** | **Trial / DOI** | **Original gold label** | **Counterfactual gold label** | **Title changed** | **Sections modified** |
| --- | --- | --- | --- | --- | --- |
| 1 | Azacitidine prolongs overall survival compared with conventional care regimens in elderly patients with low bone marrow blast count acute myeloid leukemia. 10.1200/JCO.2009.23.8329 | POSITIVE | NEGATIVE | yes | title, results, conclusion |
| 2 | Capecitabine Versus Active Monitoring in Stable or Responding Metastatic Colorectal Cancer After 16 Weeks of First-Line Therapy: Results of the Randomized FOCUS4-N Trial. 10.1200/JCO.21.01436 | POSITIVE | NEGATIVE | no | results, conclusion |
| 3 | Sequential combination of gemtuzumab ozogamicin and standard chemotherapy in older patients with newly diagnosed acute myeloid leukemia: results of a randomized phase III trial by the EORTC and GIMEMA consortium (AML-17). 10.1200/JCO.2013.49.0771 | NEGATIVE | POSITIVE | no | results, conclusion |
| 4 | Intensified platinum therapy is an ineffective strategy for improving outcome in pediatric patients with advanced hepatoblastoma. 10.1200/JCO.2005.02.6013 | NEGATIVE | POSITIVE | yes | title, results, conclusion |

**Supplementary Table 1**. Overview of included examples.

# Example 1. POSITIVE to NEGATIVE

| **Journal and date** | J Clin Oncol; 2/1/10 |
| --- | --- |
| **DOI** | 10.1200/JCO.2009.23.8329 |
| **Original gold label** | POSITIVE |
| **Counterfactual gold label** | NEGATIVE |
| **Counterfactual sections modified** | title, results, conclusion |

**Original title**

Azacitidine prolongs overall survival compared with conventional care regimens in elderly patients with low bone marrow blast count acute myeloid leukemia.

**Counterfactual title**

Azacitidine does not prolong overall survival compared with conventional care regimens in elderly patients with low bone marrow blast count acute myeloid leukemia.

**Original abstract**

PURPOSE: In a phase III randomized trial, azacitidine significantly prolonged overall survival (OS) compared with conventional care regimens (CCRs) in patients with intermediate-2- and high-risk myelodysplastic syndromes. Approximately one third of these patients were classified as having acute myeloid leukemia (AML) under current WHO criteria. This analysis compared the effects of azacitidine versus CCR on OS in this subgroup. PATIENTS AND METHODS: Patients were randomly assigned to receive subcutaneous azacitidine 75 mg/m(2)/d or CCR (best supportive care [BSC] only, low-dose cytarabine (LDAC), or intensive chemotherapy [IC]). RESULTS: Of the 113 elderly patients (median age, 70 years) randomly assigned to receive azacitidine (n = 55) or CCR (n = 58; 47% BSC, 34% LDAC, 19% IC), 86% were considered unfit for IC. At a median follow-up of 20.1 months, median OS for azacitidine-treated patients was 24.5 months compared with 16.0 months for CCR-treated patients (hazard ratio = 0.47; 95% CI, 0.28 to 0.79; P = .005), and 2-year OS rates were 50% and 16%, respectively (P = .001). Two-year OS rates were higher with azacitidine versus CCR in patients considered unfit for IC (P = .0003). Azacitidine was associated with fewer total days in hospital (P < .0001) than CCR. CONCLUSION: In older adult patients with low marrow blast count (20% to 30%) WHO-defined AML, azacitidine significantly prolongs OS and significantly improves several patient morbidity measures compared with CCR.

**Counterfactual abstract**

PURPOSE: In a phase III randomized trial, azacitidine significantly prolonged overall survival (OS) compared with conventional care regimens (CCRs) in patients with intermediate-2- and high-risk myelodysplastic syndromes. Approximately one third of these patients were classified as having acute myeloid leukemia (AML) under current WHO criteria. This analysis compared the effects of azacitidine versus CCR on OS in this subgroup. PATIENTS AND METHODS: Patients were randomly assigned to receive subcutaneous azacitidine 75 mg/m(2)/d or CCR (best supportive care [BSC] only, low-dose cytarabine (LDAC), or intensive chemotherapy [IC]). RESULTS: Of the 113 elderly patients (median age, 70 years) randomly assigned to receive azacitidine (n = 55) or CCR (n = 58; 47% BSC, 34% LDAC, 19% IC), 86% were considered unfit for IC. At a median follow-up of 20.1 months, median OS for azacitidine-treated patients was 16.2 months compared with 16.0 months for CCR-treated patients (hazard ratio = 0.97; 95% CI, 0.62 to 1.52; P = .89), and 2-year OS rates were 18% and 16%, respectively (P = .67). Two-year OS rates were not higher with azacitidine versus CCR in patients considered unfit for IC (P = .42). Azacitidine was not associated with fewer total days in hospital (P = .58) than CCR. CONCLUSION: In older adult patients with low marrow blast count (20% to 30%) WHO-defined AML, azacitidine does not prolong OS or improve patient morbidity measures compared with CCR.

# Example 2. POSITIVE to NEGATIVE

| **Journal and date** | J Clin Oncol; 11/20/21 |
| --- | --- |
| **DOI** | 10.1200/JCO.21.01436 |
| **Original gold label** | POSITIVE |
| **Counterfactual gold label** | NEGATIVE |
| **Counterfactual sections modified** | results, conclusion |

**Original title**

Capecitabine Versus Active Monitoring in Stable or Responding Metastatic Colorectal Cancer After 16 Weeks of First-Line Therapy: Results of the Randomized FOCUS4-N Trial.

**Counterfactual title**

Capecitabine Versus Active Monitoring in Stable or Responding Metastatic Colorectal Cancer After 16 Weeks of First-Line Therapy: Results of the Randomized FOCUS4-N Trial.

**Original abstract**

PURPOSE: Despite extensive randomized evidence supporting the use of treatment breaks in metastatic colorectal cancer (mCRC), they are not universally offered to patients despite improvements in quality of life without detriment to overall survival (OS). FOCUS4-N was set up to explore the impact of oral maintenance therapy in patients who are responding to first-line therapy. METHODS: FOCUS4 was a molecularly stratified trial program that registered patients with newly diagnosed mCRC. The FOCUS4-N trial was offered to patients in whom a targeted subtrial was unavailable or biomarker tests failed. Patients were randomly assigned using a 1:1 ratio between maintenance capecitabine and active monitoring (AM). The primary outcome was progression-free survival (PFS) with secondary outcomes including OS toxicity and tolerability. RESULTS: Between March 2014 and March 2020, 254 patients were randomly assigned (127 to capecitabine and 127 to AM) across 88 UK sites. Baseline characteristics were balanced. There was strong evidence of efficacy for PFS (hazard ratio = 0.40; 95% CI, 0.21 to 0.75; P < .0001), but no significant improvement in OS (hazard ratio, 0.93; 95% CI, 0.69 to 1.27; P = .66) was observed. Compliance with treatment was good, and toxicity from capecitabine versus AM was as expected with grade ≥ 2 fatigue (25% v 12%), diarrhea (23% v 13%), and hand-foot syndrome (26% v 3%). Quality of life showed little difference between the groups. CONCLUSION: Despite strong evidence of disease control with maintenance therapy, OS remains unaffected and FOCUS4-N provides additional evidence to support the use of treatment breaks as safe management alternatives for patients who are stable or responding to first-line treatment for mCRC. Capecitabine without bevacizumab may be used to extend PFS in the interval after 16 weeks of first-line therapy.

**Counterfactual abstract**

PURPOSE: Despite extensive randomized evidence supporting the use of treatment breaks in metastatic colorectal cancer (mCRC), they are not universally offered to patients despite improvements in quality of life without detriment to overall survival (OS). FOCUS4-N was set up to explore the impact of oral maintenance therapy in patients who are responding to first-line therapy. METHODS: FOCUS4 was a molecularly stratified trial program that registered patients with newly diagnosed mCRC. The FOCUS4-N trial was offered to patients in whom a targeted subtrial was unavailable or biomarker tests failed. Patients were randomly assigned using a 1:1 ratio between maintenance capecitabine and active monitoring (AM). The primary outcome was progression-free survival (PFS) with secondary outcomes including OS toxicity and tolerability. RESULTS: Between March 2014 and March 2020, 254 patients were randomly assigned (127 to capecitabine and 127 to AM) across 88 UK sites. Baseline characteristics were balanced. There was no evidence of efficacy for PFS (hazard ratio = 0.93; 95% CI, 0.69 to 1.27; P = .66), and no significant improvement in OS (hazard ratio, 0.93; 95% CI, 0.69 to 1.27; P = .66) was observed. Compliance with treatment was good, and toxicity from capecitabine versus AM was as expected with grade ≥ 2 fatigue (25% v 12%), diarrhea (23% v 13%), and hand-foot syndrome (26% v 3%). Quality of life showed little difference between the groups. CONCLUSION: Maintenance capecitabine did not improve PFS compared with active monitoring, and OS remained unaffected. FOCUS4-N provides additional evidence to support the use of treatment breaks as safe management alternatives for patients who are stable or responding to first-line treatment for mCRC. Capecitabine without bevacizumab should not be used routinely to extend PFS in the interval after 16 weeks of first-line therapy.

# Example 3. NEGATIVE to POSITIVE

| **Journal and date** | J Clin Oncol; 12/10/13 |
| --- | --- |
| **DOI** | 10.1200/JCO.2013.49.0771 |
| **Original gold label** | NEGATIVE |
| **Counterfactual gold label** | POSITIVE |
| **Counterfactual sections modified** | results, conclusion |

**Original title**

Sequential combination of gemtuzumab ozogamicin and standard chemotherapy in older patients with newly diagnosed acute myeloid leukemia: results of a randomized phase III trial by the EORTC and GIMEMA consortium (AML-17).

**Counterfactual title**

Sequential combination of gemtuzumab ozogamicin and standard chemotherapy in older patients with newly diagnosed acute myeloid leukemia: results of a randomized phase III trial by the EORTC and GIMEMA consortium (AML-17).

**Original abstract**

PURPOSE: This randomized trial evaluated the efficacy and toxicity of sequential gemtuzumab ozogamicin (GO) and standard chemotherapy in older patients with newly diagnosed acute myeloid leukemia (AML). PATIENTS AND METHODS: Patients (n = 472) age 61 to 75 years were randomly assigned to induction chemotherapy with mitoxantrone, cytarabine, and etoposide preceded, or not, by a course of GO (6 mg/m(2) on days 1 and 15). In remission, patients received two consolidation courses with or without GO (3 mg/m(2) on day 0). The primary end point was overall survival (OS). RESULTS: The overall response rate was comparable between the two arms (GO, 45%; no GO, 49%), but induction and 60-day mortality rates were higher in the GO arm (17% v 12% and 22% v 18%, respectively). With median follow-up of 5.2 years, median OS was 7.1 months in the GO arm and 10 months in the no-GO arm (hazard ratio, 1.20; 95% CI, 0.99 to 1.45; P = .07). Other survival end points were similar in both arms. Grade 3 to 4 hematologic and liver toxicities were greater in the GO arm. Treatment with GO provided no benefit in any prognostic subgroup, with the possible exception of patients age < 70 years with secondary AML, but outcomes were significantly worse in the oldest age subgroup because of a higher risk of early mortality. CONCLUSION: As used in this trial, the sequential combination of GO and standard chemotherapy provides no benefit for older patients with AML and is too toxic for those age ≥ 70 years.

**Counterfactual abstract**

PURPOSE: This randomized trial evaluated the efficacy and toxicity of sequential gemtuzumab ozogamicin (GO) and standard chemotherapy in older patients with newly diagnosed acute myeloid leukemia (AML). PATIENTS AND METHODS: Patients (n = 472) age 61 to 75 years were randomly assigned to induction chemotherapy with mitoxantrone, cytarabine, and etoposide preceded, or not, by a course of GO (6 mg/m(2) on days 1 and 15). In remission, patients received two consolidation courses with or without GO (3 mg/m(2) on day 0). The primary end point was overall survival (OS). RESULTS: The overall response rate was comparable between the two arms (GO, 49%; no GO, 45%), and induction and 60-day mortality rates were similar between the GO and no-GO arms (12% v 17% and 18% v 22%, respectively). With median follow-up of 5.2 years, median OS was 10 months in the GO arm and 7.1 months in the no-GO arm (hazard ratio, 0.83; 95% CI, 0.69 to 0.99; P = .04). Other survival end points were consistent with an OS benefit in the GO arm. Grade 3 to 4 hematologic and liver toxicities were greater in the GO arm. Treatment with GO provided benefit across prognostic subgroups, with the greatest effect observed in patients age < 70 years with secondary AML; outcomes were not significantly worse in the oldest age subgroup, with no excess risk of early mortality. CONCLUSION: As used in this trial, the sequential combination of GO and standard chemotherapy improves overall survival for older patients with AML, with increased hematologic and liver toxicity but without an excess of early mortality, including in patients age ≥ 70 years.

# Example 4. NEGATIVE to POSITIVE

| **Journal and date** | J Clin Oncol; 6/20/06 |
| --- | --- |
| **DOI** | 10.1200/JCO.2005.02.6013 |
| **Original gold label** | NEGATIVE |
| **Counterfactual gold label** | POSITIVE |
| **Counterfactual sections modified** | title, results, conclusion |

**Original title**

Intensified platinum therapy is an ineffective strategy for improving outcome in pediatric patients with advanced hepatoblastoma.

**Counterfactual title**

Intensified platinum therapy is an effective strategy for improving outcome in pediatric patients with advanced hepatoblastoma.

**Original abstract**

PURPOSE: The INT-0098 Intergroup Liver Tumor Study demonstrated no statistically significant differences in event-free and overall survival between patients randomized to treatment with either cisplatin + fluorouracil + vincristine (C5V) or cisplatin + doxorubicin. Results from this and other therapeutic trials suggested that cisplatin was the most active agent against hepatoblastoma. To increase the platinum dose-intensity, a novel regimen was developed alternating carboplatin and cisplatin (CC) every 2 weeks. The P9645 study was designed to compare the risk of treatment failure for patients with stage III/IV hepatoblastoma randomized to either C5V or CC. METHODS: C5V was given according to INT-0098 and CC consisted of carboplatin at 700 mg/m2 on day 0 (560 mg/m2 after two cycles) followed by cisplatin 100 mg/m2 on day 14. Granulocyte colony-stimulating factor was used after each CC cycle. All patients received four to six cycles of chemotherapy. RESULTS: From the time the study was opened until the time that random assignment was halted, 56 patients received CC and 53 patients received C5V. The 1-year event-free survival was 37% for patients receiving CC and 57% for those receiving C5V (P = .017). Patients randomly assigned to CC required more blood product support. As a result of a semiannual review by the Children's Oncology Group Data and Safety Monitoring Committee, random assignment was discontinued after 3 years of enrollment because the projected improvement in long-term outcome associated with CC was statistically excluded as a possible outcome of this trial. CONCLUSION: Intensification of therapy by alternating platinum analogs increased the risk of adverse outcome in children with unresectable or metastatic hepatoblastoma.

**Counterfactual abstract**

PURPOSE: The INT-0098 Intergroup Liver Tumor Study demonstrated no statistically significant differences in event-free and overall survival between patients randomized to treatment with either cisplatin + fluorouracil + vincristine (C5V) or cisplatin + doxorubicin. Results from this and other therapeutic trials suggested that cisplatin was the most active agent against hepatoblastoma. To increase the platinum dose-intensity, a novel regimen was developed alternating carboplatin and cisplatin (CC) every 2 weeks. The P9645 study was designed to compare the risk of treatment failure for patients with stage III/IV hepatoblastoma randomized to either C5V or CC. METHODS: C5V was given according to INT-0098 and CC consisted of carboplatin at 700 mg/m2 on day 0 (560 mg/m2 after two cycles) followed by cisplatin 100 mg/m2 on day 14. Granulocyte colony-stimulating factor was used after each CC cycle. All patients received four to six cycles of chemotherapy. RESULTS: From the time the study was opened until the time that random assignment was halted, 56 patients received CC and 53 patients received C5V. The 1-year event-free survival was 57% for patients receiving CC and 37% for those receiving C5V (P = .017). Patients randomly assigned to CC required more blood product support. As a result of a semiannual review by the Children's Oncology Group Data and Safety Monitoring Committee, random assignment was discontinued after 3 years of enrollment because the projected improvement in long-term outcome associated with CC was statistically confirmed as a likely outcome of this trial. CONCLUSION: Intensification of therapy by alternating platinum analogs improved outcome in children with unresectable or metastatic hepatoblastoma, despite increased requirements for blood product support.

# Supplement part 2: Additional input conditions

These supplementary analyses evaluate the three additional input conditions: abstract only, title + DOI, and counterfactual title only. Performance is evaluated against the original gold label for abstract only and title + DOI, and against the inverted counterfactual label for counterfactual title only. Only valid POSITIVE/NEGATIVE outputs are included in accuracy and F1 calculations.

| **Condition** | **Model** | **Valid predictions (%)** | **Accuracy (95% CI)** | **F1 Score (95% CI)** |
| --- | --- | --- | --- | --- |
| Abstract only | GPT-5.2 | 100.0 | 0.97 (0.95-0.99) | 0.97 (0.95-0.99) |
| Abstract only | Gemini 3 Flash | 98.8 | 0.97 (0.95-0.99) | 0.97 (0.95-0.99) |
| Abstract only | Claude Opus 4.5 | 99.6 | 0.97 (0.95-0.99) | 0.97 (0.95-0.99) |
| Title + DOI | GPT-5.2 | 100.0 | 0.86 (0.81-0.90) | 0.85 (0.81-0.90) |
| Title + DOI | Gemini 3 Flash | 98.0 | 0.92 (0.88-0.95) | 0.92 (0.88-0.95) |
| Title + DOI | Claude Opus 4.5 | 100.0 | 0.82 (0.77-0.87) | 0.82 (0.77-0.87) |
| Counterfactual title only | GPT-5.2 | 100.0 | 0.23 (0.18-0.28) | 0.22 (0.17-0.28) |
| Counterfactual title only | Gemini 3 Flash | 97.6 | 0.15 (0.10-0.19) | 0.15 (0.10-0.19) |
| Counterfactual title only | Claude Opus 4.5 | 100.0 | 0.25 (0.19-0.30) | 0.24 (0.19-0.30) |

**Supplementary Table 2**. Performance under additional reviewer-requested input conditions.

| **Model** | **Subset** | **Valid paired trials** | **Prediction flips** | **Flip rate (%)** |
| --- | --- | --- | --- | --- |
| GPT-5.2 | All trials | 250 | 22 | 8.8 |
| GPT-5.2 | Counterfactual title changed | 13 | 10 | 76.9 |
| GPT-5.2 | Counterfactual title unchanged | 237 | 12 | 5.1 |
| Gemini 3 Flash | All trials | 242 | 22 | 9.1 |
| Gemini 3 Flash | Counterfactual title changed | 13 | 11 | 84.6 |
| Gemini 3 Flash | Counterfactual title unchanged | 229 | 11 | 4.8 |
| Claude Opus 4.5 | All trials | 250 | 21 | 8.4 |
| Claude Opus 4.5 | Counterfactual title changed | 13 | 10 | 76.9 |
| Claude Opus 4.5 | Counterfactual title unchanged | 237 | 11 | 4.6 |

**Supplementary Table 3**. Prediction flip rate between original title-only and counterfactual-title-only prompts. The title-changed subset contains the 13 trials for which the counterfactual edit substantively modified the title.

# References

1. Fenaux P, Mufti GJ, Hellström-Lindberg E, et al. Azacitidine prolongs overall survival compared with conventional care regimens in elderly patients with low bone marrow blast count acute myeloid leukemia. J Clin Oncol. 2010;28(4):562-569. doi: 10.1200/JCO.2009.23.8329.
2. Adams RA, Fisher DJ, Graham J, et al. Capecitabine versus active monitoring in stable or responding metastatic colorectal cancer after 16 weeks of first-line therapy: results of the randomized FOCUS4-N trial. J Clin Oncol. 2021;39(33):3693-3704. doi: 10.1200/JCO.21.01436.
3. Amadori S, Suciu S, Stasi R, et al. Sequential combination of gemtuzumab ozogamicin and standard chemotherapy in older patients with newly diagnosed acute myeloid leukemia: results of a randomized phase III trial by the EORTC and GIMEMA consortium (AML-17). J Clin Oncol. 2013;31(35):4424-4430. doi: 10.1200/JCO.2013.49.0771.
4. Malogolowkin MH, Katzenstein H, Krailo MD, et al. Intensified platinum therapy is an ineffective strategy for improving outcome in pediatric patients with advanced hepatoblastoma. J Clin Oncol. 2006;24(18):2879-2884. doi: 10.1200/JCO.2005.02.6013.
